# Colony size buffers interactions between neonicotinoid exposure and cold stress in bumblebees

**DOI:** 10.1098/rspb.2023.0555

**Published:** 2023-07-26

**Authors:** August C. Easton-Calabria, Jessie A. Thuma, Kayleigh Cronin, Gigi Melone, Madalyn Laskowski, Matthew A. Y. Smith, Cassandra L. Pasadyn, Benjamin L. de Bivort, James D. Crall

**Affiliations:** ^1^ Department of Entomology, University of Wisconsin-Madison, Madison, WI 53706, USA; ^2^ Department of Organismic and Evolutionary Biology, Harvard University, Cambridge, MA 02138, USA; ^3^ Center for Brain Science, Harvard University, Cambridge, MA 02138, USA; ^4^ Department of Biology, Tufts University, Medford, MA 02155-5801, USA

**Keywords:** bees, social behaviour, neonicotinoid, temperature, automated tracking

## Abstract

Social bees are critical for supporting biodiversity, ecosystem function and crop yields globally. Colony size is a key ecological trait predicted to drive sensitivity to environmental stressors and may be especially important for species with annual cycles of sociality, such as bumblebees. However, there is limited empirical evidence assessing the effect of colony size on sensitivity to environmental stressors or the mechanisms underlying these effects. Here, we examine the relationship between colony size and sensitivity to environmental stressors in bumblebees. We exposed colonies at different developmental stages briefly (2 days) to a common neonicotinoid (imidacloprid) and cold stress, while quantifying behaviour of individuals. Combined imidacloprid and cold exposure had stronger effects on both thermoregulatory behaviour and long-term colony growth in small colonies. We find that imidacloprid's effects on behaviour are mediated by body temperature and spatial location within the nest, suggesting that social thermoregulation provides a buffering effect in large colonies. Finally, we demonstrate qualitatively similar effects in size-manipulated microcolonies, suggesting that group size *per se*, rather than colony age, drives these patterns. Our results provide evidence that colony size is critical in driving sensitivity to stressors and may help elucidate mechanisms underlying the complex and context-specific impacts of pesticide exposure.

## 1. Introduction

Bees and other insect pollinators are critical for supporting biodiversity [1], crop productivity [2,3] and human wellbeing [4], and there is mounting concern over declines of bee populations and associated risks to the ecosystem services they provide. Diverse stressors negatively affect bee health, including climate change [5], disease and pathogens [6], land use change [7], and agrochemical exposure [8]. Neonicotinoid pesticides in particular have strong, sublethal impacts on the behaviour and physiology of bees that can contribute to population declines [9,10]. In addition to their direct effects, neonicotinoids can also have strong synergies with other environmental stressors, including other agrochemicals [11], nutrition [12], temperature [13,14] and pathogens [15].

A significant challenge in understanding the real-world impacts of neonicotinoids is identifying their effects on group behaviour and, in turn, how collective colony dynamics may mitigate neonicotinoids' impacts [16]. Despite representing a minority of bee species, social bees are of particular ecological and agricultural importance and include dominant crop-pollinating species [17]. Colony size is a critical ecological trait for social insects with widespread effects on colony behaviour and performance [18–20] and may play a role in buffering the effects of stressors [21]. While the highly social corbiculate bees (i.e. honeybees (Apini) and stingless bees (Meliponini)) have large perennial colonies, bumblebee (*Bombus* spp.) colonies are initiated by a solitary queen at the beginning of each colony cycle. The role of colony size in modulating stressors and their interactions may be especially important for bees with such strong seasonal variation in sociality. After nest-founding, colonies can grow rapidly to several hundred workers before initiating seasonal reproduction [22], such that the size of a bumblebee colony may vary across more than two orders of magnitude within just a few months.

While many theoretical studies predict a positive effect of colony size on robustness to stressors (i.e. positive density dependence, also known as Allee effects [23–26]), empirical evidence is limited and mixed. Some previous work has shown lower sensitivity to pesticides in social bee taxa (i.e. honeybees) relative to solitary bees [27], while other studies have found evidence for stronger negative effects of pesticide exposure on social (versus solitary) bee taxa [28]. Sociality may increase sensitivity to stressors in certain contexts, such as increased social transmission of pathogens, or sensitivity to disruption of social communication [16,29]. Understanding mechanisms underlying positive density dependence is especially important for characterizing (and predicting) real-world impacts of complex environmental stressors—and their interactions—on social bees. Yet evidence for such mechanisms is particularly lacking for non-*Apis* bees, with most evidence for mechanisms of positive density dependence coming from theoretical studies focusing on *A. mellifera* [25,30].

Here, we experimentally test the role of colony size and growth in modulating sensitivity to stressors and their interactions, focusing on two critical environmental stressors: neonicotinoid exposure and temperature. Neonicotinoid pesticides have widespread sublethal effects on bumblebees (including foraging [31], learning [32,33] and social behaviour [14]) that can lead to impaired colony growth and fitness [27,34]. Temperature is also strongly linked to performance of bumblebee colonies and population health [5]; social temperature homeostasis within the nest has facilitated adaptation of bumblebees to cold climates and is critical for successful colony growth [35–37]. Neonicotinoid exposure and temperature stress also have important synergistic effects [14,38,39], including disruption of social thermoregulation [14].

To assess the role of colony size in modulating the synergistic effects of temperature and neonicotinoids, we exposed bumblebee colonies to combinations of imidacloprid and cold temperatures across a range of colony sizes, using both captive, queenright colonies of three bumblebee species (*Bombus impatiens*, *B. bimaculatus* and *B. griseocollis*) and size-manipulated microcolonies (*Bombus impatiens*). Integrating automated behavioural and thermal tracking with long-term growth monitoring, we tested the hypotheses that (i) combined exposure to cold and neonicotinoids have stronger synergistic effects on adaptive thermoregulatory behaviour in small colonies; (ii) combined, transient exposure to cold and neonicotinoids also have stronger synergistic effects on colony growth in small colonies; (iii) worker location within the nest modulates the effects of neonicotinoid exposure; and (iv) thermoregulatory feedbacks underlie greater robustness to stressor interactions in large colonies.

## 2. Methods

### (a) Queenright colony experiments

#### (i) Specimen collection and nest rearing

**Figure 1.**
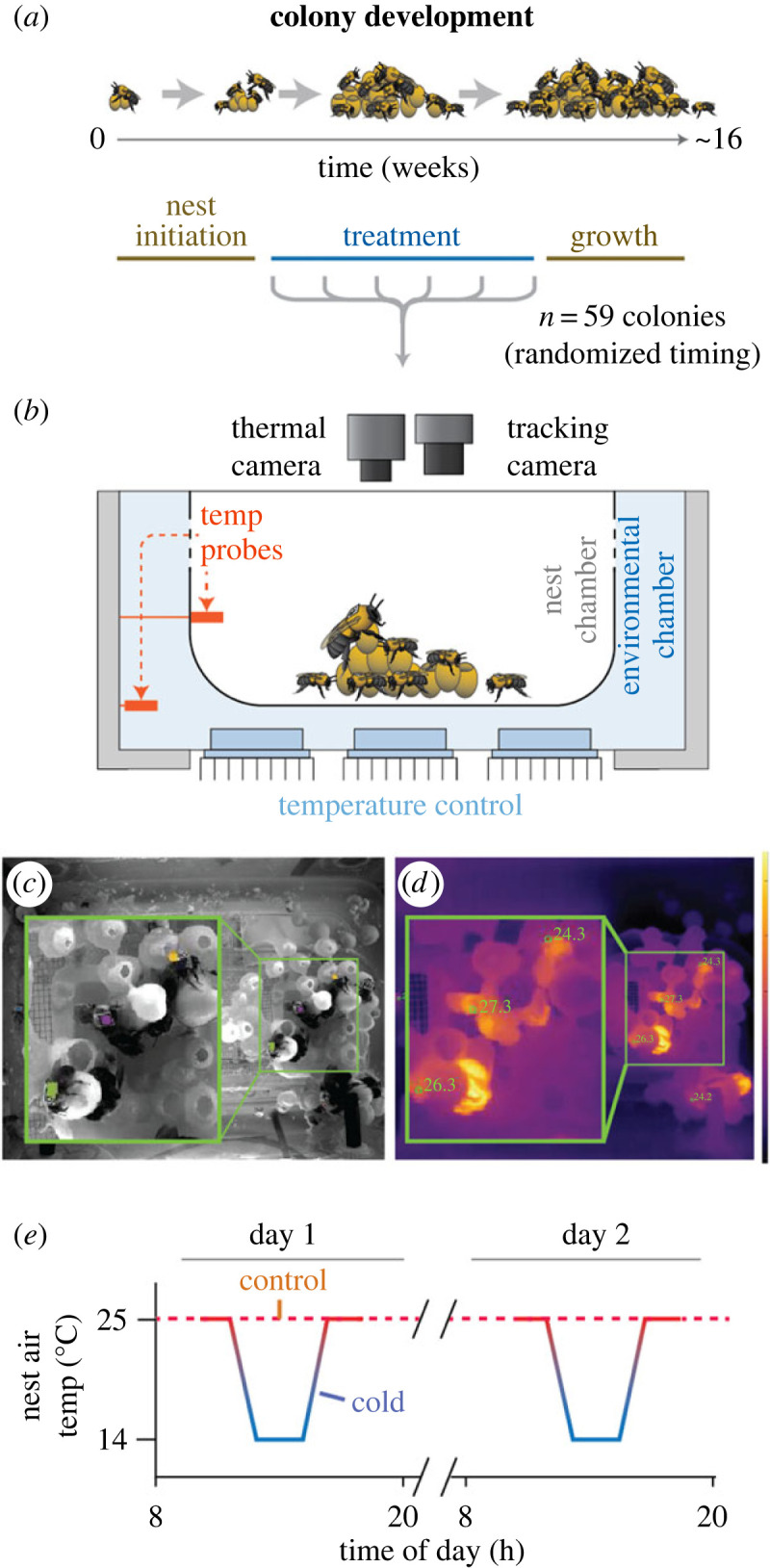
Experimental design and tracking system for queenright colony experiments. Schematic diagrams for (*a*) colony development and treatment timing, (*b*) arena for tracking and temperature control. (*c,d*) Example images were taken simultaneously from the tracking (*c*) and thermal (*d*) cameras. Inset boxes show a magnified region of the brood structure. Tracked coordinates of tagged bees were detected in the tracking camera image and overlaid in the thermal image. (*e*) Cold exposure regime.

We collected data from 59 experimental bumblebee colonies, of three different species: *B. impatiens* (*n* = 29), *B. bimaculatus* (*n* = 15) and *B. griseocollis* (*n* = 15). These colonies were reared in the laboratory under controlled environmental conditions (26°C, 60% RH, 14 : 10 L : D cycle) from wild-caught queens collected in spring (14 April–8 May) of 2019 in Massachusetts. Queens were initially housed in small (15.2 cm × 7.6 cm) plastic chambers with *ad libitum* access to nectar and fresh pollen (electronic supplementary material, figure S1). On 25 June, the sixty colonies with the most advanced colony development (i.e. emerged workers in most colonies, or well-developed larvae and pupae in some cases) were transferred to custom colony growth chambers (electronic supplementary material, figure S1; L × W × H: 19.5 cm × 13.0 cm × 11.0 cm) made from Sterilite® tupperware containers, modified to include screened ventilation holes on walls, nectar access on the container bottom, and fitted with a custom clear acrylic imaging top (figure 1).

Colonies were then assigned to one of 15 experimental blocks containing four roughly size-matched colonies. Size-matching was achieved by ordering colonies by visually estimated size (number of bees), then assigning colonies sequentially to blocks of four. Given variation across nests in queen capture date, time to nest initiation, and initial growth rate, colonies varied in initial colony size at this stage from a single queen in the smallest block to approximately 20 workers (based on visual estimation at the time and confirmed by reviewing recorded videos) in the largest block.

#### (ii) Experimental treatments

Twice weekly, one experimental block was randomly selected for exposure to a 48 h stress treatment. Each colony was exposed only once during the experiment to a single 48 h stress treatment. Within each block, each colony was randomly assigned to each of four experimental treatment groups: (i) cold stress (2 h at air temperature approximately 14°C within the nest once daily for 2 days, see below) only, (ii) imidacloprid exposure only (10 ppb in sucrose solution for 48 h), (iii) combined imidacloprid and cold stress or (iv) control (pure sucrose solution and unmanipulated nest temperatures). 10 ppb was chosen for the imidacloprid concentration to reflect the upper end of the realistic distribution of concentrations in nectar, which can vary widely across crops and application regimes [40,41]. The timing of treatment was randomized during seven weeks of colony development, such that colonies were exposed at varying colony stages and sizes (figure 1, colony size range at time of treatment = 1–184 bees). After the 48 h exposure period, colonies were returned to standard culture conditions to continue colony growth until final colony census (mean colony experiment length ± s.d. ≈ 15.4 ± 1.4 weeks from first brood emergence until census). All treatments occurred within a period of 48 days (9 July–26 August 2019)

#### (iii) Tagging and automated behavioural tracking

Prior to experimental trials, each colony was anaesthetized with CO_2_ and all adult bees (workers and queens) were removed from the nest. Each bee was tagged with a unique BEEtag barcode [42] using cyanoacrylate glue. After tagging, all bees were returned to the nest, which was placed in an imaging platform that separately housed four colonies. Colonies had *ad libitum* access to fresh pollen and nectar throughout the experiment (including during treatment). The imaging platform was a modified version of one previously used for parallel monitoring of bumblebee colonies [14,43] and consisted of a camera mounted to a Cartesian gantry system, with the camera position controlled using Matlab scripts and a SmoothieBoard stepper driver. Video sequences (2 min, approx. 1.5 Hz) were recorded from each colony in succession once every approximately 9–10 min for approximately 48 h using a Point Grey monochrome camera (Grasshopper 3, 3000 × 4096 px), mounted alongside a high-resolution thermal camera (see below). Due to a combination of intermittent hardware failures and colonies with no available tracking data (e.g. colonies in which no bees were tracked due to wax-covered tags, etc.), behavioural and thermal imaging data were unavailable for 17 of 59 colonies. Hardware failures did not affect temperature manipulation or neonicotinoid exposure, so these colonies were included in models of long-term colony growth (described below). Excluded colonies did not differ significantly from included colonies in size (Wilcoxon rank sum test, *W* = 275, *p* = 0.25).

Positions and identities of each visible tag were tracked within each frame using previously established methods [14,44]. Structural elements of the nest (including developing larvae and brood, food storage pots and nectar reservoirs) were manually mapped for each colony using custom Matlab scripts, following previously established techniques [44]. In brief, images of each colony's nest structure were created by digitally removing bees through median pixel background estimation. Centroids of wax pots and brood were then manually annotated.

#### (iv) Temperature manipulation

Air temperature was controlled in each colony separately (figure 1*a*). The housing around each colony was insulated with closed-cell foam padding on the sides and bottom. Three digital temperature probes (DS18B20) were placed in each colony, two within the inner nest chamber, and one in the outer environmental chamber (i.e. the space between the nest arena and the outer chamber walls; figure 1*b*). Temperature readings were taken from all probes from each colony roughly every 20 s via Matlab scripts and an Arduino Uno microcontroller. Temperature was controlled in the outer environmental chamber of each nest via Peltier thermoelectric heat pumps (TEC1-12710) placed on the bottom of each colony. Peltiers were connected to heat sinks and air-circulating fans on both faces (i.e. one within and one outside the chamber; figure 1*b*), with control signals generated via a custom PID controller implemented in Matlab. In cold-exposed colonies, temperature manipulations were implemented over the course of 4.5 h. Air temperatures in the outer environmental chambers were initially brought from room temperature to 24°C for 10 min before ramping down to approximately 10°C over the course of an hour (0.25°C min^−1^). Air temperatures were then maintained at approximately 10°C in the outer chamber for 2 h before ramping back up to 24°C over the course of an hour (also 0.25°C min^−1^) and finally held at 24°C for 20 min, after which nest temperatures were not actively controlled. Cold-stressed colonies were exposed to this cold-stress period once a day (twice total over the 48 h monitoring period), with the initiation of the 4.5 h cold exposure period occurring at the same time across all cold-treated colonies, and corresponding to the overnight period in their L : D cycle (given higher behavioural effects occurring overnight, see [14]). Air-circulating fans were run continuously in all colonies over the entire monitoring period so that any behavioural impacts of air flow or vibration would be constant over the experiment. Mean air temperatures within the nest chamber were slightly higher (mean across colonies = 14.3°C) than the (actively controlled) temperatures within the outer environmental chamber during the period of minimum (approx. 10°C) exposure.

#### (v) Thermal imaging and calibration

Videos were taken from a high-resolution thermal camera (FLIR A65, 640 × 512 resolution, 50 mK sensitivity) simultaneously with the tracking camera (see above). Absolute pixel temperature values from the thermal camera were calibrated against the digital temperature probes (covered in black masking tape to maximize emissivity) inserted within the nest, the locations of which were manually annotated for each colony. This internal calibration was performed for each thermal camera frame separately and provided highly accurate temperature measurements, verified against a secondary temperature probe (electronic supplementary material, figure S2). To ensure that any differences in emissivity between materials (i.e. probes, wax, tags and bees) resulted in negligible differences in their measured temperature across the temperature gradient, we confirmed the mean accuracy of temperature readings taken through each material (mean difference from the reference temperature probes: 0.089°C; wax: 0.090°C, bees: 0.14°C; electronic supplementary material, figure S2).

#### (vi) Thermal image registration and thermal tracking

Body temperature of individual bees and brood temperature were quantified from these corrected thermal images by mapping coordinates from the tracking camera to the thermal camera. To account for the three-dimensional architecture of the nest structure, a non-rigid b-Splines deformation between manually annotated calibration coordinates in both the tracking and thermal images [45] was implemented in Matlab and calculated for each colony separately. This transformation was used to locate the position of tracked tags in thermal images. Body temperature was quantified by taking the mean temperature within a radius of 20 pixels (approx. 6 mm) of the tag centroid. This averaged value showed strong correlation with single point measurements, and this automated approach showed strong agreement with body temperatures estimated manually from thermal images (electronic supplementary material, figure S3).

#### (vii) Colony demography

Initial colony size was quantified at the time of treatment by counting workers, males and queens separately. If present, reproductives (males and newly emerged queens) were removed from the colony before further experimental treatment to mimic gynes leaving the colony. Final colony size and demography data were collected beginning 15 days after final experimental manipulations had been completed (41.4 ± 15.9 days between treatment and census across colonies). Total colony productivity was quantified as the total number of individuals (workers and reproductives) produced by the colony, including dead bees present at the time of final census.

### (b) Queenless microcolony experiments

#### (i) Experimental design

Thirty-one microcolonies were created from three source colonies by CO_2_ anaesthetizing and removing either four (for ‘small’ microcolonies, *N* = 21) or 16 (for ‘large’ microcolonies, *N* = 10) workers from mature *Bombus impatiens* colonies (Koppert Biological), along with developing brood. The workers were chosen randomly and were only replaced if they showed visible signs of old age (e.g. missing hair, missing legs and torn wings). Worker : brood ratio was kept constant at 2 : 1 for both colony sizes. Pupae and well-differentiated larvae were used for brood. Each microcolony was housed in a custom imaging chamber made from laser-cut acrylic and three-dimensional-printed (PLA) plastic components and had *ad libitum* access to pollen and nectar. The microcolony experiments were conducted in three rounds, using one source colony per round. For each round, microcolonies of the same size were randomly split between either control or neonicotinoid pesticide exposure (10 ppb imidacloprid provided *ad libitum* in nectar) treatments. Microcolonies were housed under controlled environmental conditions (26°C, 60% RH) for approximately 48 h before being subjected to cold stress.

#### (ii) Tagging and automated behavioural tracking

Each worker was tagged using a unique ArUco tag (a Python-based fiducial marker-tracking system [46]) with cyanoacrylate glue. Tags were printed on waterproof plastic paper. The imaging chamber (L × W × H: 152 mm × 152 mm × 188 mm) used a Raspberry Pi 4b single-board computer and an associated Raspberry Pi High Quality Camera (4056 × 3040 px) to image the colonies at approximately 3.75 Hz for 20 s every 2 min. The infrared filter of the camera was removed and near-infrared (approx. 840 nm) lights were used to illuminate the microcolonies. Within-nest air temperature was monitored using a T-type thermocouple and an MCC 134 Thermocouple Measurement DAQ HAT for Raspberry Pi.

The identities and positions of each ArUco tag were tracked within each frame using the ArUco Python library. Briefly, adaptive thresholding is applied to each image and any tag-shaped contours are identified; the candidate tags are then confirmed and the identity and location stored, or candidate is discarded.

#### (iii) Cold stress

Each cold-stress trial lasted for approximately 5 h and 10 min. Each microcolony was moved into an environmental control room set to 22°C between 2.5 and 3 h before it underwent cold stress. The temperature was then ramped down to 10°C over the course of 1 h and 5 min (0.18°C min^−1^) and was held there for 3 h before ramping back up to 22°C over 1 h and 5 min.

### (c) Behavioural quantification

#### (i) Queenright experiments

We calculated 11 separate behavioural metrics for each bee following previous work [44]. Individual behavioural metrics were then aggregated for individual bees for each 2 min video trial (figure 1; electronic supplementary material, S3). Interaction rates for brood and wax pots reflect the strength of spatial overlap between individual bees' spatial distribution within the nest and all brood and wax pots, respectively, and accounts for potential interactions with multiple nest elements simultaneously. These were calculated by multiplying spatial probability distributions of each bee by the number of identified nest elements in each spatial bin within the nest.

Spatial correlation to nest-mates was calculated for each pair of bees within each video sequence, using the same two-dimensional spatial distribution described above. For each bee, the mean correlation to all other nest-mates was then calculated. Mean interaction rate was calculated as the mean rate of physical interaction (less than 1 cm between centroids) across all nest-mates (i.e. number of contacts/bee/frame), averaged for each bee for a video sequence. Network centrality was calculated as degree centrality, or the number of unique interactions with nest-mates. Moving speed was calculated as the mean speed (pixels/frame) when the bee was moving, defined as frames where speed exceeded an activity ‘threshold’ (defined using the bimodal distribution of frame-wise speed values, as in [44]).

Distance to the centre was calculated as the mean distance from the bee's centroid to the nest centre (defined socially as the mean position of all bees within a video), averaged across all frames. Minimum distance to the closest brood and wax pot was calculated as the median value for each bee across all frames. Median distance to all brood and wax pots and minimum distance to the closest brood and wax pot elements were calculated as the median across all frames within a video sequence for each bee.

The dimensionality of these behavioural metrics was reduced using principal components analysis (PCA) implemented in R [47], with input variables scaled and centred. The first principal component (37% of variance explained) captured spatial centrality within the nest (electronic supplementary material, figure S4). Principal components were transformed into an approximately normal distribution using the Yeo–Johnson transformation for subsequent analyses.

#### (ii) Queenless microcolony experiments

For queenless microcolonies, a unique set of behavioural variables were selected specifically for their likely association with spatial centrality as in the above analysis (distance to other bees, minimum distance to brood, brood contact rate, distance to social centre of the nest and number of physical contacts with nest-mates). PCA of these variables yielded a first principal component that explained 69% of variance and is also referred to as ‘spatial centrality’ (electronic supplementary material, figure S5).

### (d) Statistical modelling and analysis

We constructed linear mixed effects models (LMMs) and generalized linear mixed effects models (GLMMs) to assess effects of treatment (categorical), colony size (continuous for queenright experiments (log_10_-transformed), categorical for queen microcolony experiments) and species (categorical) on behaviour and colony growth, respectively, using the ‘lme4’ package in R [48]. All LMMs (for both queenright colonies and microcolony experiments) included individual bee identity nested within colony (both categorical), and experimental block (categorical) as random effects. LMMs for queenright colony experiments also included caste (worker versus queen), and day of treatment (factor, i.e. first versus second day of the 48 h stress period) as random effects.

For LMMs of behaviour in queenright colony experiments where multiple species were present, the most complex models assessed included three-way interaction terms between colony size (at treatment), imidacloprid exposure and temperature exposure, as well as a two-way interaction term between nest temperature and species (to assess evidence for potential differential response to cold temperature between species), as fixed effects.

For the GLMM of treatment effects on colony growth (outcome variable: total colony productivity, Poisson family model and log link), the most complex models assessed included a three-way interaction term between colony size (at treatment, continuous), cold treatment (categorical) and imidacloprid exposure (categorical) as fixed effects, and experimental block (categorical), position in temperature control chamber (categorical), and species (categorical, with random intercepts and also random slopes for colony size at treatment, to account for species-specific difference in colony growth) as random effects. To account for variability in total colony productivity associated with time available for growth after treatment, we constructed additional models that included the number of days between treatment and final census as a continuous, fixed effect. These models showed qualitatively similar effects, but failed to properly converge, and so models excluding this variable are presented here. Results for these alternative models are available in the code and data supplement.

Model fits were assessed using the DHARMa package in R [49], and models were compared and selected using AIC, preferring simpler models except where AIC comparison demonstrated significant model improvement (ΔAIC > 4). Significance of individual terms of the best-fitting models (based on AIC) was calculated using the lmerTest package [50] with degrees of freedom estimated via Satterthwaite's method.

Generalized additive models (GAMs) were implemented to assess the effects of body temperature on proximate behavioural metrics using the ‘gam’ and ‘bam’ functions in the ‘mgcv’ package in R [51], and included colony, individual identity, and species as random effects.

## 3. Results

### (a) Active behavioural responses to cold exposure in queenright colonies

**Figure 2.**
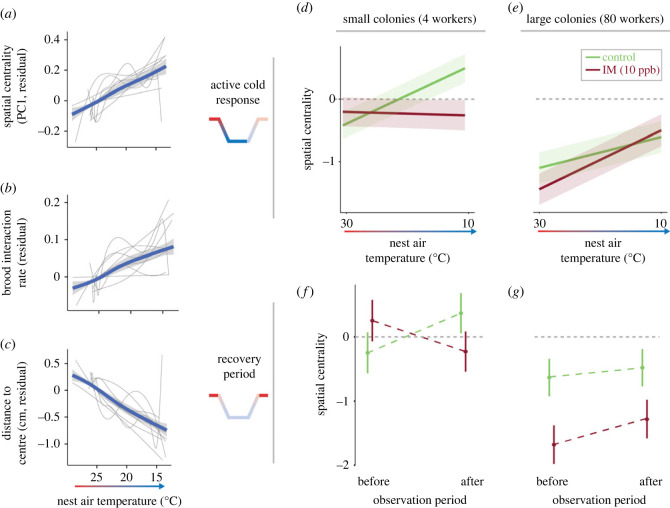
Behavioural impacts of cold exposure in queenright colonies. (*a–c*) Effects of decreasing temperature on residual (*a*) spatial centrality (PC1), (*b*) brood interaction rate and (*c*) distance to the nest centre. Residuals are estimated from a LMM including only individual and colony as random effects. In (*a–c*), grey lines show smoothed (via gam) estimated responses for *n* = 9 separate colonies exposed to cold and fed imidacloprid-free sucrose. Thick blue lines and shaded regions show smoothed mean estimate (via gam) across all nine colonies. (*d,e*) Effects of nest temperature on spatial centrality in small ((*d*), colony size = 4) and large ((*e*), colony size = 80) colonies during active cold exposure (*n* = 9718 observations from *n* = 1017 individuals across *n* = 41 colonies in models shown in *d,e*). (*f,g*) Effects of cold treatment on spatial centrality after versus before exposure in small (*f*) and large (*g*) colonies. Colonies exposed to imidacloprid shown in red (IM, 10 ppb) and control colonies in green (*n* = 2155 observations from *n* = 463 individuals across *n* = 20 colonies in models shown in *f,g*). Responses shown in (*d–g*) are estimated from a LMM. While colony size is a continuous variable in models, modelled responses are shown or two representative colony sizes (*n* = 4 workers in *d*,*f*, *n* = 80 workers in *e*,*g*) to illustrate differential responses across colony size. Estimated effects and 50% CIs are shown as solid lines and shaded regions, respectively, in (*a*,*b*). Solid markers and vertical lines, respectively, in (*c*,*d*). See text and electronic supplementary material, tables S1 and S2, for model details.

We quantified a total of 123 325 two-minute behavioural sequences from 1292 individually tagged bees across 42 colonies in our queenright colony experiment using high-resolution individual tracking (BEEtag [42]) and thermal cameras (1.5 Hz) during the 48 h stress-exposure treatments (figure 1*b–d*). Dimensionality reduction of behavioural tracking metrics using PCA revealed a primary axis (principal component 1, 37.9% of total variance explained) associated with spatial centrality within the nest (electronic supplementary material, figure S3), consistent with previous work in bumblebees [44] and with the known importance of spatial structure for social insect behaviour [20,52,53]. This first principal component is referred to as ‘spatial centrality’ hereafter. Spatial centrality increased with decreasing temperature (figure 2*a*) in cold-exposed colonies fed imidacloprid-free sucrose solution, corresponding to increased rates of interaction with the brood (figure 2*b*) [54] and decreased distance from the nest centre (figure 2*c*). Increased spatial centrality is consistent with adaptive thermoregulatory clustering and increased incubation in response to cold in bumblebees [54,55].

We quantified the effects of temperature on spatial centrality during the active cooling phase of each exposure period (from 24°C to approximately 14°C, 0.2°C min^−1^ ramp rate). AIC model selection provided evidence for significant differences between species in spatial centrality scores, but did not support a two-way interaction between nest temperature and species, suggesting that active thermoregulatory clustering responses were similar across the three study species (electronic supplementary material, table S1). The best-supported model also included a three-way interaction between nest air temperature, colony size and imidacloprid exposure; small, imidacloprid-free colonies showed robust, active clustering (higher spatial centrality, PC1) in response to cold, whereas this response was strongly reduced in small, imidacloprid-exposed colonies (figure 2*d,e*; *n* = 9718 observations; *Δ*AIC to next best model = 8.7; LMM: [imidacloprid] × [air temperature] × [log_10_(colony size)]: d.f. = 7190, *t* = −3.99, *p* = 6.62 × 10^−5^).

Next, we quantified lingering behavioural impacts of transient cold exposure by comparing spatial centrality before and after cold exposure, during periods of baseline air temperature within the nest (approx. 24°C; figure 2*f,g*), focusing exclusively on colonies that had been exposed to cold (*n* = 21 colonies). Similar to active responses to cold, the best-supported model included species as a fixed effect, but did not include an interaction between observation period (before versus after cold) and species, suggesting no evidence for species differences in behavioural recovery after cold exposure (electronic supplementary material, table S2). The best-supported model also included a three-way interaction between monitoring period (before versus after exposure), colony size and imidacloprid exposure (figure 2*f,g*; *n* = 2,155 observations; ΔAIC to next best model = 21.2; LMM: [imidacloprid] × [pre versus post] × [log_10_(colony size)]: d.f. = 1900, *t* = 4.70, *p* = 2.75 × 10^−6^). Small, control-fed colonies increased spatial centrality after cold exposure, while small, imidacloprid-exposed colonies decreased spatial centrality (figure 2*f*). However, this effect was not present in large colonies, where spatial centrality increased to a small degree in both control and imidacloprid colonies after cold exposure (relative to pre-exposure levels; figure 2*g*).

### (b) Body temperature dependence of imidacloprid's proximate behavioural effects

**Figure 3.**
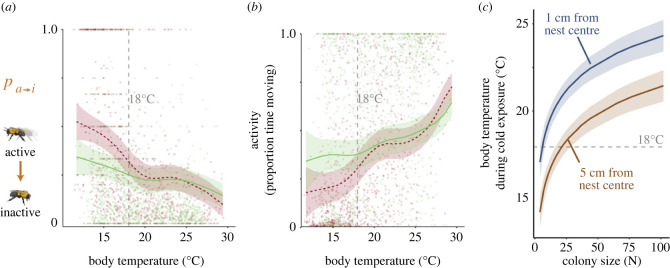
Body temperature-dependent impacts of imidacloprid. (*a*) Markovian probability of transitioning from active (a, moving) to inactive (i, immobile) and (*b*) overall activity (proportion of time moving) versus bee body temperature during cold exposure (*n* = 9839 observations total from 1066 individual bees each for *a*,*b*). (*c*) The effect of colony size on mean body temperature during cold exposure (nest air temperature less than 15°C), at a distance of 1 cm and 5 cm from the nest centre (*n* = 2110 observations total from *n* = 384 individual bees). Lines and shaded regions show mean estimates and 95% CIs (*a,b*) or 50% CIs (*c*), from a GAM in (*a*,*b*) and LMM in (*c*). Estimates for control (sucrose-fed) and imidacloprid-exposed colonies are shown in green and red, respectively, in (*a,b*).

To investigate how temperature stress and imidacloprid interact to affect individual behaviour, we quantified the effect of body temperature on individual locomotor behaviour. First, body temperature of freely behaving individual workers within each colony was quantified from calibrated thermal images (figure 1*c,d*). We then quantified the effect of body temperature on Markovian transition probabilities between activity states (i.e. moving or not moving), following previous work [14]. We found that the probability of transitioning from active (moving centroid) to inactive (stationary centroid) decreased at higher body temperatures. Imidacloprid exposure significantly increased that probability predominantly at bee body temperatures less than 18°C (figure 3*a*). This effect corresponded to reduced overall activity (i.e. time spent moving) in imidacloprid-treated bees at lower body temperatures (figure 3*b*).

Both colony size and location within the nest significantly affected body temperature. Mean body temperature was higher in larger colonies (figure 3*c*; LMM: log_10_(colony size); residual d.f. = 17.0, *t* = 4.1, *p* = 0.0008) and decreased with distance from the nest centre (figure 3*c*; LMM: distance to nest centre; residual d.f. = 2509, *t* = −20.2, *p* = 2 × 10^−16^), consistent with collective thermoregulation occurring primarily on brood located near the nest centre.

### (c) Individual variation in spatial location and neonicotinoid sensitivity

**Figure 4.**
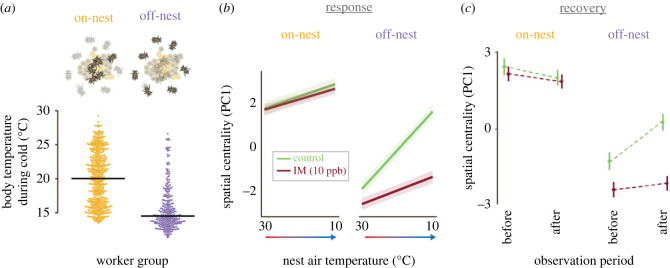
Differential impacts across worker groups in moderately sized colonies (*n* < 30). (*a*) Body temperature during cold exposure for workers located on the nest (left) and off the nest (right) prior to cold treatment. Points are individual bees, and data are pooled from control and imidacloprid-treated colonies and across species (*n* = 605 observations from *n* = 89 individuals). Black lines indicate median value of each group. (*b*) Effects of temperature on spatial centrality in response to decreased nest temperatures by worker initial position (on versus off nest). (*c*) Change in spatial centrality after cold exposure by worker initial position (on versus off nest). Shaded regions in (*b*) and vertical solid bars in (*c*) represent 50% CIs.

To further explore the impacts of body temperature and its intersection with individual behavioural variation within small bumblebee colonies [44], we compared behavioural responses between workers located on or off the nest in small and moderately sized colonies (less than 30 workers). We isolated smaller colonies for this analysis to focus on colony size ranges where stronger effects occurred and to reduce model complexity; qualitative results were not sensitive to selection of this colony size cutoff (data not shown). Workers initially located off-nest (greater than 2 cm from the nearest brood) had lower body temperatures during cold exposure than workers initially located on the nest (less than 1 cm to nearest brood) in colonies (figure 4*a*; LMM: d.f. = 86.1, *t* = −5.63, 2.2 × 10^−7^), while imidacloprid exposure did not have a significant effect on worker body temperature during cold (LMM; d.f. = 9.1, −0.18, *p* = 0.86). Off-nest workers specifically showed behavioural impairment after imidacloprid exposure. In small, control-fed colonies, off-nest workers showed active clustering (higher spatial centrality) in response to cold, but this response was strongly impaired in imidacloprid-exposed colonies (figure 4*b*; *n* = 1151 observations; ΔAIC to next best model = 12.1; LMM: [nest temperature] × [imidacloprid] × [on- versus off-nest]: d.f. = 1110, *t* = 2.41, *p* = 0.016). By contrast, the spatial centrality of workers initially located on-nest was unaffected by imidacloprid (figure 4*b*). Similarly, the effects on behavioural recovery after cold were much stronger among off-nest workers than on-nest workers (figure 4*c*; *n* = 569 observations; ΔAIC to next best model = 7.7; LMM: [before versus after exposure] × [imidacloprid] × [on- versus off-nest]: d.f. = 514, *t* = −2.26, *p* = 0.024).

### (d) Impacts on colony growth

**Figure 5.**
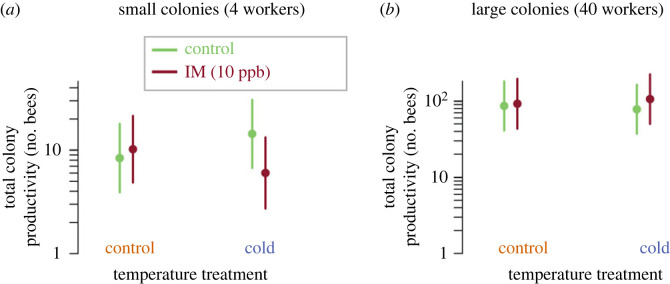
Impacts of colony size (at treatment) and treatment condition on total colony productivity. Modelled total colony productivity (total no. of bees, alive and dead, at final census) estimates are shown for initial (at treatment) colony sizes of 4 (*a*) and 40 (*b*) workers under different treatment conditions. Filled circles and vertical solid lines represent estimated mean and 95% CI for estimates of colony productivity (*n* = 55 colonies total across *n* = 3 species). Forty workers were chosen as a representative larger colony size here (rather than 80 as in others figures) given the exclusion of colonies initially (at treatment) larger than 40 workers in this analysis.

We found strong evidence for a three-way interaction between imidacloprid exposure, cold stress and colony size (at exposure) on total colony productivity at the end of the experiment; combined exposure to cold and imidacloprid had stronger negative effects on total colony productivity when exposure occurred at smaller colony sizes (figure 5; electronic supplementary material, figure S6; *n* = 55 observations; ΔAIC to next best model = 7.7; GLMM, Poisson distribution, [imidacloprid] × [cold] × [log_10_(colony size)]: residual d.f. = 45, *z* = 3.63, *p* = 2 × 10^−4^). Colonies with only a single individual (i.e. queen) and colonies larger than 40 total bees at treatment were excluded from this analysis (in the latter case to minimize effects of colonies approaching maximum capacity), although inclusion of these colonies had no qualitative effect on results (electronic supplementary material, figure S7). Alternative models quantifying effects on final number of living bees and on colony reproductive output (queens and males produced) showed qualitatively similar effects (electronic supplementary material, figure S7).

### (e) Behavioural impairment in size-manipulated microcolonies

**Figure 6.**
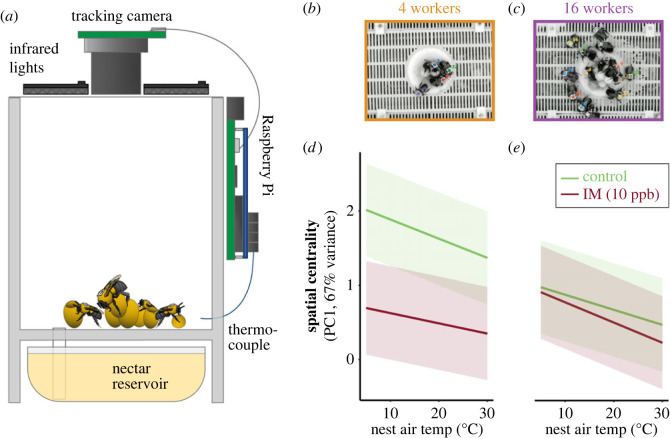
Effects of air temperature on spatial centrality in size-manipulated microcolonies of *Bombus impatiens*. (*a*) Schematic diagram of microcolony tracking arena. (*b,c*) Sample images with example tracking of uniquely identified individual workers are shown for small ((*b*), *n* = 4 workers) and large ((*c*), *n* = 16 workers) microcolonies. Effects of nest temperature on spatial centrality for small (*d*) and large (*e*) colonies, shown separately for control (green) and imidacloprid-fed (red) colonies. Solid lines and shaded regions show mean estimates and 50% CIs from a LMM.

A total of 117 877 20 s behavioural sequences were recorded from *n* = 237 individual workers across all *Bombus impatiens* microcolonies (*n* = 31 microcolonies total; four-worker microcolonies: *n* = 21 total, *n* = 10 sucrose-fed, *n* = 11 imidacloprid in sucrose; sixteen-worker microcolonies: *n* = 10 total, *n* = 5 sucrose-fed, *n* = 5 imidacloprid in sucrose) using the Raspberry Pi-based tracking system (figure 6*a–c*). Spatial centrality increased at lower temperatures, qualitatively similar to the behaviour of workers in queenright colonies (electronic supplementary material, figure S8). Imidacloprid reduced spatial centrality more strongly overall in small microcolonies (figure 6*d,e*) and more strongly impaired active responses to cold in small (*n* = 4 worker) microcolonies (figure 6*d,e*; *n* = 41 316 observations; ΔAIC to next best model = 15.8; LMM: [imidacloprid] × [nest temperature] × [colony size]: d.f. = 4.1 × 10^4^, *t* = −4.0, *p* = 5.9 × 10^−5^).

## 4. Discussion

We found combined cold and neonicotinoid exposure have the strongest effects on both behaviour and colony growth in small colonies (figures 2, 5, 6), consistent with the hypothesis of increased robustness in larger colonies (i.e. positive density dependence, or Allee effects). Colony size and stressor interactions have been separately recognized as critically important for understanding pollinator health [56–58]; here we show that colony size can also modulate interactions between stressors. Smaller colonies also showed higher sensitivity when group size was experimentally manipulated (figure 6), providing strong evidence that group size *per se* (rather than colony stage, age or development) is an important factor driving these effects.

Colony size may specifically mitigate interactions between cold and neonicotinoids by altering their proximate impacts on behaviour. This represents a distinct mechanism from other proposed drivers of positive density dependence within social insect colonies (such as buffering of individual exposure, including shielding of reproductives [21], or creating differential consequences of individual impairment [30,59]). Collective thermoregulation may be particularly important in the scaling of sensitivity with colony size; we found that imidacloprid's effects on locomotor behaviour depend on body temperature, with the probability of becoming inactive rising primarily at lower body temperature (figure 3*a,b*). This finding is consistent with recent evidence for interactive effects of temperature and neonicotinoid exposure in bumblebees (*Bombus terrestris*) [60]. Combined with lower average body temperature during cold exposure in smaller colonies (figure 3*c*), this suggests that workers in smaller colonies are more likely to encounter body temperatures at which imidacloprid's proximate effects emerge.

The strong effect of worker location within the nest on responses to cold and neonicotinoid exposure provides further support for an important role of body temperature. In small colonies, on-nest workers maintained higher body temperatures during cold exposure than off-nest workers (figure 4*a*). Imidacloprid exposure strongly impaired behaviour of off-nest workers during active response to cooling and recovery, but had comparatively little effect on warm-bodied workers on the nest (figure 4*b,c*). Previous work has shown that workers vary substantially in spatial location within the nest [59], and that spatial location has important effects on task switching [44]. Our results here suggest that this individual variation in spatial location within the nest also drives differential behavioural responses to neonicotinoid exposure across workers.

These results may also help shed light on mechanisms underlying previously observed spatial effects within bumblebee colonies. Specifically, in previous work [14,26], we have found that the effects of imidacloprid on locomotor state transition probabilities are dependent on location within the nest. The effects of imidacloprid on locomotor transition rates are stronger when bees are located off the nest, compared to on the nest [14]. Our results here suggest that spatial differences in body temperature within the nest (with higher body temperatures in proximity to the nest centre; figures 3*c*, 4*a*) may be the proximate mechanism underlying this observed effect.

A potential alternative mechanism for the observed patterns of strong impacts in small colonies is the scaling of social contact rate, which positively correlates with colony size (electronic supplementary material, figure S9) and can modulate the effects of imidacloprid [14,26]. Consistent with this, higher contact rates are predicted to increase activity in larger colonies, which we also observed here (electronic supplementary material, figure S9). However, previous modelling has suggested that worker density has a relatively weak effect [26] on social buffering compared to location (and hence body temperature), which—in combination with our findings here—suggests that body temperature and spatial location have a stronger effect than rates of social contact. However, reduced social interaction rates in smaller colonies may play an additional role that could exacerbate the impacts of cold and imidacloprid interactions.

It is possible that, under imidacloprid exposure, key tasks (such as incubation) are maintained by a core of warm-bodied workers in the short term, while longer term task reallocation is impaired. In particular, recruitment of off-nest workers appears to play an important role in the collective response to cold in control colonies (figure 4*b,c*), and its impairment by imidacloprid could result in long-term impairment of colony task allocation and performance. Consistent with this, reduced brood temperature in small, imidacloprid-exposed colonies occurred after (but not during) cold exposure (electronic supplementary material, figures S10 and S11, and text). These findings underscore the importance of not only direct, immediate effects of stressors, but also indirect and potentially delayed effects [61] as an important but understudied factor.

Our results are consistent with a functional link between impaired behaviour and reduced long-term colony growth in neonicotinoid-exposed bumblebee colonies. Long-term effects of altered behaviour on colony growth could arise from greater impacts *during* exposure, such as impaired thermoregulation and brood care during the 48 h of exposure. Such acute behavioral effects could have lasting impacts on the brood, including impaired growth and development (or in extreme cases, brood death). Alternatively, long-term reductions in colony growth could arise from lingering behavioural effects that extend beyond the acute exposure period. For example, workers exposed to imidacloprid and cold stress in small colonies could show stronger long-term impairment of behaviour in the days and weeks following exposure. Future experiments monitoring behaviour for extended periods beyond acute exposure would be helpful to assess the importance of these effects. Likewise, experimentally manipulating colony size while separately controlling larval versus adult worker exposure would help to further disentangle growth effects arising from impaired behaviour versus those arising from direct larval exposure. Experiments manipulating colony size while monitoring long-term growth effects would also help disentangle the importance of colony size from potential colony-level phenotypic variation in growth rate, which may be partially confounded in our queenright experiments here.

Our finding that the combination of cold and imidacloprid exposure has stronger impacts in small colonies has important ecological implications. First, while the *severity* of exposure is often a primary focus, the *timing* of exposure may be equally relevant. In developing bumblebee colonies, identical exposure to cold stress and neonicotinoids will have more negative effects when experienced during early colony development. Yet empirical evidence addressing how agrochemical exposures fluctuate over time in natural environments is limited. For stressors with nonlinear impacts on organismal performance (as is true for both neonicotinoid pesticides and temperature), understanding the impacts of fluctuating, dynamic stress exposure is critical for predicting real-world impacts (per Jensen's inequality [62]). In the case of neonicotinoids, exposure patterns are likely to fluctuate strongly depending on application practices, environmental fates (e.g. transmission into soil, groundwater and non-crop flowers [63,64]) and flowering phenology.

The range of temperature stresses experienced by bumblebee colonies within real nests, especially across species, is a similarly important knowledge gap. Our results did not provide evidence for significant differences across species (*Bombus impatiens, B. bimaculatus* and *B. griseocollis*) in social thermoregulatory responses when exposed to cold (electronic supplementary material, tables S1 and S2). However, bumblebees show significant variation in nesting location both within and between species [65], including between the three species studied here; *B. impatiens* nests primarily (although not exclusively) underground, while *B. griseocollis* primarily nests on the soil surface, and *B. bimaculatus* shows more mixed nest selection [65]. Field data suggest the experimental temperature conditions employed here are within the realistic range experienced within bumblebee nests (electronic supplementary material, figure S12 and text) in the natural range and habitat of these species. However, while mean temperatures are similar, temperature fluctuations depend strongly on nesting depth (electronic supplementary material, figure S12), suggesting that surface-nesting species may be more susceptible to temperature stress and its interactions with neonicotinoid exposure than below-ground nesting species.

Taken together, our results highlight the critical role of colony size in robustness and resilience to anthropogenic and environmental stressors. Improved understanding of the linkages between individual behaviour and colony dynamics—which may be facilitated by automated, scalable behavioural tracking—will help elucidate the complex and context-dependent effects of stressors on bees and other social insects.

## Data Availability

All accompanying data and analysis code are available at: https://github.com/Crall-Lab/socialScaling. Additional information is provided in the electronic supplementary material [66].
